# Generation of an Oocyte-Specific Cas9 Transgenic Mouse for Genome Editing

**DOI:** 10.1371/journal.pone.0154364

**Published:** 2016-04-27

**Authors:** Linlin Zhang, Jiankui Zhou, Jinxiong Han, Bian Hu, Ningning Hou, Yun Shi, Xingxu Huang, Xin Lou

**Affiliations:** 1 Model Animal Research Center, MOE Key Laboratory of Model Animal for Disease Study, Nanjing University, Nanjing, China; 2 School of Life Science and Technology, ShanghaiTech University, Shanghai, China; Institute of Zoology, Chinese Academy of Sciences, CHINA

## Abstract

The CRISPR/Cas9 system has been developed as an easy-handle and multiplexable approach for engineering eukaryotic genomes by zygote microinjection of Cas9 and sgRNA, while preparing Cas9 for microinjection is laborious and introducing inconsistency into the experiment. Here, we describe a modified strategy for gene targeting through using oocyte-specific Cas9 transgenic mouse. With this mouse line, we successfully achieve precise gene targeting by injection of sgRNAs only into one-cell-stage embryos. Through comprehensive analysis, we also show allele complexity and off-target mutagenesis induced by this strategy is obviously lower than Cas9 mRNA/sgRNA injection. Thus, injection of sgRNAs into oocyte-specific Cas9 transgenic mouse embryo provides a convenient, efficient and reliable approach for mouse genome editing.

## Introduction

Genetic modified mice are essential tools for scientists to gain the necessary understanding of gene function and diseases, and to discover improved methods to prevent, diagnose and treat diseases. In recent years, a series of programmable nuclease-based genome editing technologies have been developed [1–4]. These strategies enable efficient gene targeting and modification, which could prominently facilitate the generation of genetic modified mouse model for biomedical research [5–8].

Of the current generation of genome editing approaches, the most rapidly developing is a type of RNA-guided endonucleases known as Cas9 from the microbial adaptive immune system CRISPR (clustered regularly interspaced short palindromic repeats) [9, 10]. In the most widely used form of this system, two elements must be introduced into and/or expressed in cells or an organism to perform genome editing: the Cas9 nuclease and a guide RNA (gRNA). Twenty nucleotides at the 5’end of the gRNA direct Cas9 to a specific target DNA site using standard RNA-DNA complementarity base-pairing rules. These target sites must lie immediately 5’ of a PAM sequence that matches the canonical form 5’-NGG. At the binding sites defined by gRNA, the HNH and RuvC-like nuclease domains on Cas9 protein cut both DNA strands, generating double-stranded breaks (DSBs) [11]. Cas9 induced DSBs can be repaired by one of at least two different pathways that operate in nearly all organisms: nonhomologous end-joining (NHEJ) and homology-directed repair (HDR). NHEJ can lead to the efficient introduction of insertion/deletion mutations (indels) of various lengths, which can disrupt the translational reading frame of a coding sequence or the binding sites of *trans*-acting factors in promoters or enhancers [12]. HDR-mediated repair can be used to introduce specific point mutations or to insert desired sequences through recombination of the target locus with exogenously supplied DNA ‘donor templates’ [11, 13, 14].

Existing strategy to generate gene-targeting mouse by CRISPY/Cas9 system need preparing Cas9 DNA, mRNA or protein, which is laborious and introducing inconsistency into the experiment. Theoretically, specifically introducing Cas9 into oocyte by transgene could simplify the procedure and boost the efficiency of gene targeting through avoiding the mosaic distribution of Cas9 protein in the early stage embryo.

In this study, we documented that the successful generation of oocyte specific expression Cas9 transgenic mouse, and the site-specific gene modification can be efficiently achieved by injection of sgRNA only into the transgenic embryos at one-cell stage. Our data also suggested this oocyte-expression-Cas9 strategy could efficiently attenuate the allele complexity. Furthermore, by careful characterization, we demonstrated off-target mutagenesis induced by this strategy is relative low.

## Materials and Methods

All the protocols involving the use of animals were in accordance with approved guidelines from the Institutional Animal Care and Use Committee of Nanjing University. All the mice were housed in Individually Ventilated Cages (IVC) in a specific pathogen-free (SPF) animal facility credited by the Association for Assessment and Accreditation of Laboratory Animals Care (AAALAC) on a 12-hours light and 12-hours dark cycle. The facility is maintained in a constant temperature and humidity environmental conditions. All animal welfare and experimental procedures are approved by the Animal Care and Use Committee of the Model Animal Research Center, Nanjing University. Every cage contain five or less mice to ensure comfortable activity space. The mice drinking water filled in independent water bottle and fodder were supplied whole day. The IVC cages were changed and cleared every week and the physical condition of the animals was monitored by veterinary department every 2 day. During the experiments, no animals died prior to the experimental endpoint. Carbon dioxide inhalation was used as the method of euthanasia for all animals utilized in this research.

### Animals

The animals used in this study were regularly maintained in an Assessment And Accreditation Of Laboratory Animal Care credited SPF animal facility on a 12-h light/dark cycle. All the protocols involving the use of animals were in accordance with approved guidelines of the Institutional Animal Care and Use Committee of the Nanjing University.

### Vector Construction and *In Vitro* Transcription

To construct the vector for generation of oocyte-specific mouse, Cas9-N-NLS-Flag-linker coding sequence was digested from pST1374-Cas9-N-NLS-Flag-linker (Addgene 44758) with endonuclease Nhe I and Age I (New England Biolabs) and sub-cloned into the digested plasmid pInsulator-Zp3-MCS which was linearized with the same endonuclease Nhe I and Age I using standard methods. Subsequently, the plasmid was linearized with I-Ceu I and then was injected into the male pronuclei of fertilized zygotes (strain C57BL/6J) using standard techniques to produce transgenic founder mice.

To construct the recombinant vector for preparation of sgRNA by *in vitro* transcription, the two complementary DNA oligos shown in S3 Table were annealed to be double-stranded and subcloned into pUC57-T7-gRNA vector as described [5]. Using the constructed recombinant vector that was completely linearized by the endonuclease Dra I as the templates, sgRNAs were produced via *in vitro* transcription using MEGAshortscript kit (Ambion) and purified using MEGAClear kit (Ambion) as described in the manuals. Using the Cas9 mRNA *in vitro* transcription vector (Addgene No. 44758) as templates, Cas9 mRNAs were produced and purified as described previously by Shen *et al*. [5].

### sgRNA Design

For the mouse Ar and NLRP3 gene, the Cas9 sites were selected with the sequence 5’-GGN(19)GG (S2 Table). The 5’dinucleotide GG ensures optimal expression from the T7 and U6 promoters.

### Reverse Transcription PCR

Mouse tissues were frozen in liquid nitrogen quickly and disrupted in TRIzol (Life Tech) with a homogenizer. Total RNA was extracted according to the manufacturer’s instructions. Remained genomic DNA in total RNA was erased with recombinant DNase I (Takara). cDNAs were reverse transcribed with HiScript II1st Strand cDNA Synthesis Kit (Vazyme) with random primer. The cDNA products were used for RT-PCR with Cas9 specific primers.

### Western Blotting

Proteins were separated by sodium dodecyl sulfate-polyacrylamide gel electrophoresis (SDS-PAGE). For immunoblotting, proteins were transferred to polyvinylidene fluoride (PVDF) membrane using an electrophoretic transfer apparatus (Bio-Rad). The membrane was blocked with 1% non-fat milk (Bio-Rad) and incubated with 1:1000 diluted primary antibody against Cas9 (AbCam) followed by 1:10,000 HRP-conjugated secondary antibodies (Sigma). Signal detection was performed using Pico West Chemiluminescent Substrate (Thermo Scientific).

### Production of Gene-Modified Mice via Zygote Injection with sgRNAs

sgRNAs were mixed at the final concentrations of 10 ng/mL. To collect oocytes, female ZP3-Cas9 transgenic mice (bodyweight of 12–14 g) were injected with HCG after 48 h treated with PMSG. 16 h after HCG injection, oocytes were collected and fertilized *in vitro*. The collected zygotes were subjected to cytoplasmic microinjection with the sgRNA mixture. Correspondingly, the zygotes obtained from C57BL/6J mouse were injected with a mixture of Cas9 mRNA and the two Ar sgRNAs. When zygotes developed into blastocyst stage embryos, the embryos were collected and investigated by PCR and T7EN1 cleavage assay.

### T7EN1 Cleavage Assay and Sequencing

Different samples were collected and digested in a lysis buffer (0.4 M NaCl, 2 mM EDTA, 1% SDS, 10 mM Tris-HCl, and 100 mg/ml Proteinase K). The genomic DNA of the sample was extracted from lysate by phenol-chloroform, and recovered by alcohol precipitation. T7EN1 cleavage assay was performed as described by Shen *et al*.[5]. Briefly, the targeted fragments were amplified by PrimerSTAR HS DNA polymerase (TaKaRa, DR010A) from the genomic DNA, then purified with a PCR cleanup kit (Axygen, AP-PCR-50). The primers for amplifying targeted fragments were listed in S3 Table. The purified PCR product was denatured and re-annealed in NEBuffer 2 (NEB) using a thermocycler. The PCR products were digested with T7EN1 (NEB, M0302L) for 30 min at 37°C and separated on a 2.5% agarose gel. The PCR products with mutations detected by T7EN1 cleavage assay were sub-cloned into T vector (Takara, D103A). For each sample, the colonies were picked up randomly and sequenced by M13F primer (5’-CGC CAG GGT TTT CCC AGT CAC GAC-3’).

### Off-Target Assay

To determine the site-specific cleavage of the CRISPR-Cas9 system *in vivo*, the potential off-target loci were searched by an open tool, SeqMap [15]. The mismatch parameter of target sequence was set as described [16]. ‘NGG’ and ‘NAG’ were chosen as PAM. The sites that have conserved 7 bp proximal to PAM with total mismatches < 5 bp and the sites with total mismatches < 4 bp were chosen as potential off-target sites for subsequent test. The selected potential off-target sites were PCR amplified using genomic DNA as templates. The PCR products were first subjected to T7EN1 cleavage assay. The potential off-target sites yielding typical cleavage bands were considered as candidates. The PCR products of the candidates were cloned and sequenced to confirm the off-target effects. The primer pairs used were listed in S4 Table.

### Statistics

Statistical analysis was performed using Prism version 5.0 (GraphPad). Results are expressed as the mean ± standard deviation (SD). Group results were compared using analysis of variance after arcsine transformation of the percentages; P < 0.05 was considered statistically significant.

## Results and Discussion

### Generation of Oocyte-Specific Cas9 Transgenic Mouse

**Fig 1:**
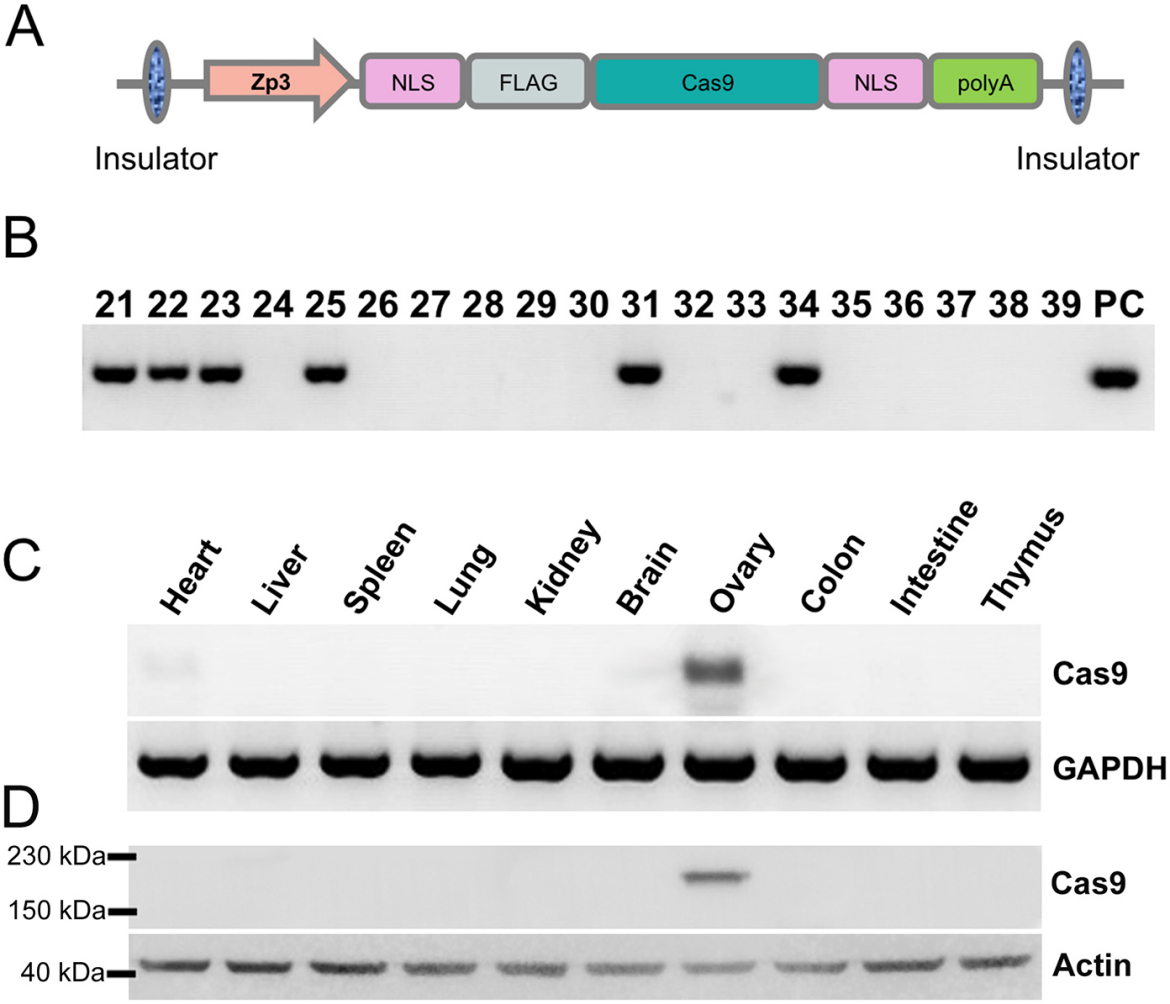
Generating a oocyte-specific Cas9 transgenic mouse line. (A) A schematic representation of the vector expressing Cas9 in oocyte. The Zp3-Cas9 plasmid encodes the Cas9 under the regulation of a 2 kb Zp3 promoter. Two nuclear localization signals (NLSs) were inserted into N and C terminals of Cas9. (B) Six transgenic founders from total 39 F0 were identified by Cas9 gene amplification by PCR. PC, positive control. (#21, #22, #23 are germ-line heritable). (C and D) RT-PCR and Western blotting confirmed the ovary specific expression of Cas9 in Zp3-Cas9 transgenic mouse.

To establish a simplified and consistent CRISPR/Cas9 system for mouse gene targeting, we sought out to generate an oocyte-specific Cas9 transgenic mouse as a tool strain. To this end, a 2.5 kb of ZP3 gene 5'-flanking sequence was incorporated to 5’ of Cas9 open reading frame on the transgene construct (Fig 1A), this fragment has been shown direct expression of interested protein to oocytes in transgenic mice [17]. A total of 338 fertilized C57BL/6J background mouse eggs were microinjected with the ZP3-Cas9 transgene and were transplanted into oviducts of outbred pseudopregnant mice. As a result, a total of 49 mice were born. PCR analysis of tail DNA was used to assess incorporation of the ZP3-Cas9 transgene into the mouse genome. 6 of the newborn animals exhibited a DNA band on agarose gels that corresponded to amplification of a segment of the Cas9 gene (Fig 1B). We bred all the positive transgenic mice for two generations, and then bred positive mice with wild type littermates over several generations. To check the possible effect of oocyte expression of Cas9 on mouse reproductive capacity, we examined little size and oocyte number in superovulated female mice. We found the ZP3-Cas9 female mice gave birth to average 7.3 pups, which is comparable to C57J/B6 mice (7.5 pups per female). Quantification results for the number of ovulated oocytes induced by superovulation in ZP3-Cas9 mouse and C57J/B6 mouse also showed no obvious difference (Table 1). Fertilization ability of oocytes was not different in these two groups. To confirm the oocyte expression of Cas9, various tissues were excised from progeny of the 3 transgenic lines, and were assayed by RT-PCR and western blotting. As shown in Fig 1C, the ZP3-Cas9 transgene mouse exhibited high level of Cas9 expression in ovaries excised from 45-day-old animals. On the other hand, all other tissues exhibited no detectable Cas9 expression.

**Table 1:** ZP3-Cas9 mouse shows normal fecundity.

Genetic background	Average litter size	Average no. of oocytes /superovulated female
ZP3-Cas9 transgene	7.3 ± 1.4*	43 ± 4.5**
C57BL/6J	7.5 ± 2.3*	48 ± 6.7**

Ten female mice were used as oocyte donors in each group. The results are expressed as mean ± SD.

*P>0.05 between C57BL/6J and zp3-Cas9 mouse

**P>0.05 between C57BL/6J and zp3-Cas9 mouse.

### Generation of Gene Targeting Mouse through sgRNA Only Injection

**Fig 2:**
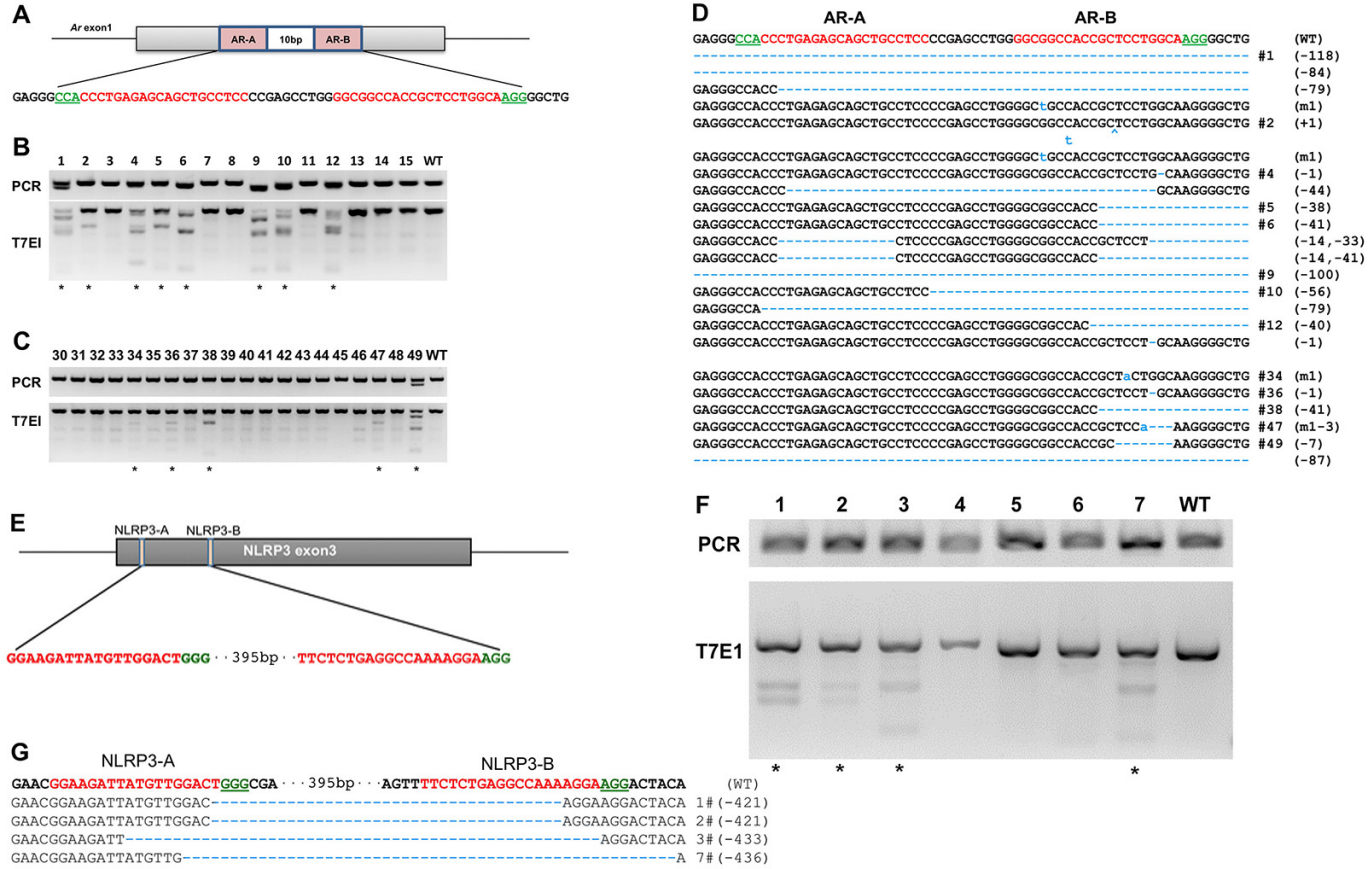
The sgRNA:Cas9-mediated modifications in Cas9 expression oocytes. (A and E) Schematic of two sgRNAs targeting the mouse *Ar* gene and NLRP3 gene. (B) PCR products of the targeted, and detection of Cas9-mediated on-target cleavage on wild-type zygotes by co-injection Cas9 mRNA and Ar sgRNAs by T7EI cleavage assay. (C and F) PCR products of the targeted, and detection of Cas9-mediated on-target cleavage on Zp3-Cas9 transgenic mouse zygotes injected with sgRNA only by T7EI cleavage assay. (D and G) Sequencing results of modified alleles in Zp3-Cas9 transgenic mouse zygotes injected with sgRNAs.

To examine the gene knockout potential of oocyte-specific Cas9 transgenic mouse, we mutated the Ar (androgen receptor) and NLRP3 (NLR family, pyrin domain containing 3) by sgRNA injection. To target Ar, we constructed two sgRNAs that bind adjacent sites on opposite strands in exon 1 of the Ar gene (Fig 2A), the efficiency of these sgRNAs has been demonstrated in previous study [18]. To target NLRP3, we constructed two sgRNAs that bind on exon 3 of the NLRP3 gene (Fig 2E). We also injected and collected fertilized wild type oocytes with Cas9 mRNA and Ar sgRNAs as control to make better assessment of gene targeting efficiency in ZP3-Cas9 mouse. After injected 188 ZP3-Cas9 transgenic embryos with the different sgRNA sets, we recovered 154 pups, the total survival rate of embryo is comparable to Cas9 mRNA/sgRNAs injection set (Table 2). Genomic DNA from the tails of the founder mice were extracted and products from PCR amplification of the target region were subjected to the T7 endonuclease 1 (T7EN) cleavage assay. Cleavage products could be detected in founder mice from both experiment sets. The results showed 26% (Ar sgRNA injected) and 31% (NLRP3 sgRNA injected) founder mouse from ZP3-Cas9 mouse showed gene targeting, which is lower than Cas9 mRNA/sgRNAs injection (41%). Using stronger oocyte specific promoter could be one possible solution for getting higher targeting efficiency. We further confirmed the mutant alleles present in the tail samples by sequencing (Fig 2C–2F and supporting information data). Similar to previous reports [5–7], introduction of sgRNA in ZP3-Cas9 mouse induced a range of deletion sizes (Fig 2D–2G and S1 Table). Since Ar gene located on X chromosome, we analyzed the targeting efficiency between two genders and no significant difference was observed (S1 Fig). These data indicate efficient *in vivo* genome editing of the ZP3-Cas9 transgenic mouse by injection of sgRNA only.

**Table 2:** sgRNA injection efficiently induces gene targeting in ZP3-Cas9 embryo.

Target Gene	Zygotes Background	Embryos Injected	Embryos Survived	Survival Rate	Targeting Efficiency
Ar	C57BL/6J	70	55	78.4%	51%
Ar	Zp3-Cas9 Tg	125	107	85.6%	26%
NLRP3	Zp3-Cas9 Tg	63	47	74.6%	31%

### Analysis of Mosaicism

It has been noticed injection of Cas9 mRNA leads a perdurance and mosaic distribution of CAS9 protein in different blastomeres, which eventually cause mosaic gene targeting [19]. This mosaicism brings about laborious genotyping and retards mouse breeding (it takes at least an extra generation time to get the gene targeting mouse since most F0 mice carry a variety of alleles). We compared the allele complexity between F0 mice derived from oocyte-expression-Cas9 strategy and routine Cas9 mRNA/sgRNAs injection. All the F0 mice derived from oocyte-expression-Cas9 strategy carry only one targeted allele except #40, while the mice from Cas9 mRNA/sgRNAs injection average carry 3 different alleles (Table 3). This indicated oocyte-expression-Cas9 strategy could attenuate the allele complexity and has the advantage to allow the researchers design a shorter breeding schedule.

**Table 3:** Oocyte-expression-Cas9 strategy efficiently attenuates the allele complexity in F0 mice.

Allele complexity in mice derived from oocyte-expression-Cas9 strategy								
F0 mouse	#25	#29	#38	#40	#64	#73	#89	Average No. of mutation
No. of mutant allele	1	1	1	2	1	1	1	1.1
Allele complexity in mice from Cas9 mRNA/sgRNAs injection								
F0 mouse	#1	#2	#6	#23	#26	#28	#29	Average No. of mutation
No. of mutant allele	4	2	3	3	4	3	2	3.0

### Analysis of Off-Targeting Effects

**Fig 3:**
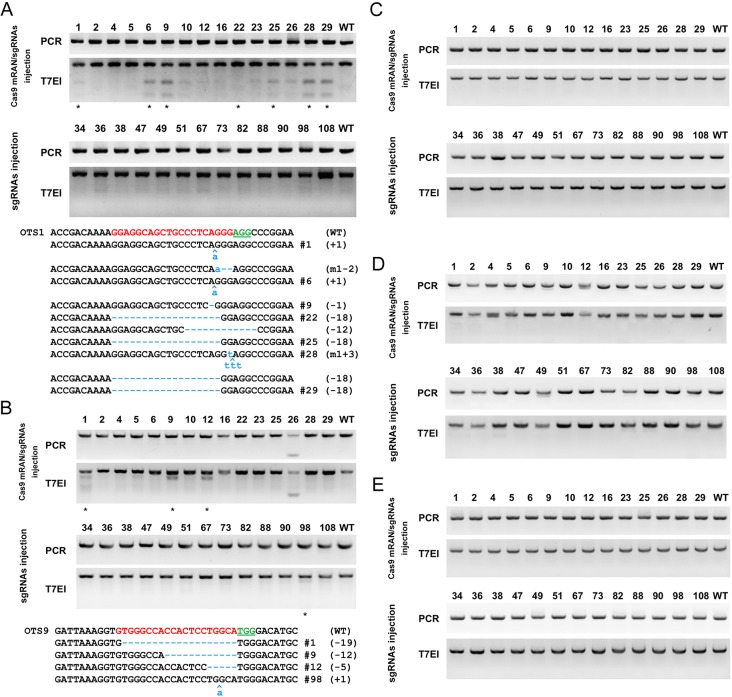
The sgRNA: Cas9-mediated off-target cleavage *in vivo*. (A) PCR products of the targeted, and detection of Cas9-mediated AR-A OTS1 off-target cleavage by T7EI cleavage assay. Lower panel, sequencing results of mutant off-target sites. (B) PCR products of the targeted, and detection of Cas9-mediated AR-B OTS9 off-target cleavage by T7EI cleavage assay. Lower panel, sequencing results of mutant off-target sites. (C to E) PCR products of the targeted, and detection of Cas9-mediated off-target cleavage by T7EI cleavage assay on OTS2, OTS3 and OTS11.

Off-target damage is of a major concern for CRISPR/Cas9 system [20–22]. To assess the specificity of this oocyte-expression-Cas9 strategy, we looked for off-target damage in *Ar* mutant founder mice. Putative off-target sites for Ar sgRNAs were identified by a scan of the entire mouse genome for all possible sites with homology to the 23 bp sequence (sgRNA + PAM), allowing for ungapped alignments with up to 5 mismatches in the sgRNA target sequences. This strategy predicted a total of 16 most potential off-target sites (OTS (off-target site) 1–8 for Ar-A sgRNA and OT 9–16 for Ar-B sgRNA) on mouse genome (Table 4). For the mutant founder mice, all the recovered OTSs were subjected to PCR amplification and T7EN1 cleavage assay. Consistent with previous report, the non-specific mutations could only be detected on OTS-1 and OTS-9 [18]. Results showed among the mutant founder mice generated from Cas9 mRNA/sgRNAs injection, 46% have genetic modification on OTS-1 and 20% have genetic modification on OTS-9. Reversely, only 1 mouse (7.7%) generated from sgRNA only injection has genetic modification on OTS-9 and no off-targeting on OTS-1 was detected. Sequencing of the PCR products confirmed the off-target cleavage events (Fig 3A and 3B). These data indicate the oocyte-expression-Cas9 strategy lead much lower off-target damage compare to Cas9 mRNA/sgRNAs injection.

**Table 4:** Putative off-target sites of Ar-A and Ar-B sgRNAs.

Position	20	19	18	17	16	15	14	13	12	11	10	9	8	7	6	5	4	3	2	1	N	G	G
Ar-A	G	G	A	G	G	C	A	G	C	T	G	C	T	C	T	C	A	G	G	G	T	G	G
OTS1	G	G	A	G	G	C	A	G	C	T	G	C	C	C	T	C	A	G	G	G	A	G	G
OTS2	G	G	A	G	G	C	A	C	C	T	G	C	A	C	T	C	A	G	G	G	T	G	G
OTS3	A	G	A	G	G	C	T	G	C	T	G	C	T	C	T	C	A	G	G	G	T	G	G
OTS4	A	G	A	G	G	C	A	G	C	T	G	G	T	C	T	C	A	A	G	G	C	G	G
OTS5	T	G	A	G	G	C	A	G	C	T	G	G	T	C	T	C	T	G	G	G	T	G	G
OTS6	T	G	G	T	G	C	A	G	C	T	G	C	T	C	T	C	A	G	G	G	A	G	G
OTS7	G	T	C	A	C	C	A	G	C	T	G	C	T	C	T	C	A	G	G	G	T	G	G
OTS8	G	G	T	C	T	C	A	G	C	T	G	C	T	C	T	C	A	G	G	G	T	G	G
Position	20	19	18	17	16	15	14	13	12	11	10	9	8	7	6	5	4	3	2	1	N	G	G
Ar-B	G	G	C	G	G	C	C	A	C	C	G	C	T	C	C	T	G	G	C	A	A	G	G
OTS9	G	T	G	G	G	C	C	A	C	C	A	C	T	C	C	T	G	G	C	A	T	G	G
OTS10	G	G	C	G	G	C	C	A	C	C	G	C	G	T	C	T	G	G	C	C	C	G	G
OTS11	G	G	C	G	G	C	C	A	C	C	C	C	T	G	C	T	G	G	C	A	C	G	C
OTS12	G	G	C	G	G	C	G	C	C	C	G	C	G	C	C	T	G	G	C	A	C	G	G
OTS13	G	G	G	G	G	C	C	A	C	C	A	C	T	C	C	T	G	G	C	A	G	G	G
OTS14	G	C	C	T	G	C	C	A	C	C	T	C	T	C	C	T	G	G	C	A	T	G	G
OTS15	G	G	C	G	G	C	C	A	C	G	G	C	T	C	C	T	G	G	C	A	G	C	T
OTS16	A	G	C	A	G	C	C	A	C	C	T	G	T	C	C	T	G	G	C	A	A	G	G

Together, these data indicated that injection of sgRNAs only into oocyte-specific Cas9 transgenic mouse embryo is a convenient, efficient and reliable approach for mouse genome editing.

## Supporting Information

S1 Fig Sex identification using PCR with a set of primers for Sry gene. (PDF)

S1 Sequencing Result Sequencing results from Zp3-Cas9 transgenic mouse injected with Ar sgRNAs. (PDF)

S2 Sequencing Result Sequencing results from C57BL/6J mouse injected with Cas9 mRNA and Ar sgRNAs. (PDF)

S1 Table Summary of mutant alleles with sgRNA:Cas9-mediated modifications in Zp3-Cas9 transgenic mouse embryo injection sgRNAs on AR locus. (PDF)

S2 Table The oligonucleotides for generating sgRNA expression. (PDF)

S3 Table The primers for genotyping, amplifying of Cas9/sgRNA targeted fragment and sex identification. (PDF)

S4 Table Primers for PCR amplification of the off-target sites. (PDF)
