# Supplementary material for: Generation of an Oocyte-Specific Cas9 Transgenic Mouse for Genome Editing
Source: PLoS One. 2016 Apr 27;11(4):e0154364. doi: 10.1371/journal.pone.0154364 (PMC4847922; doi:10.1371/journal.pone.0154364)
Supplement: S1 Fig — (PDF) [file pone.0154364.s001.pdf]

A

B

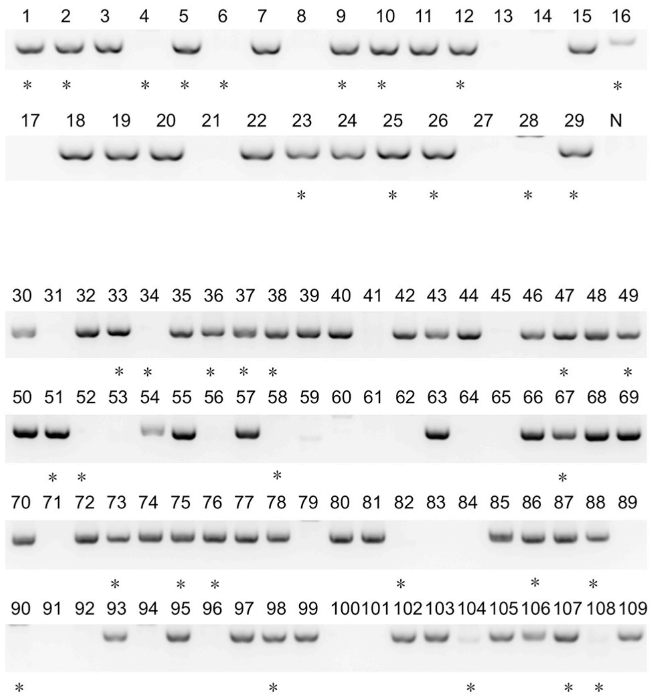

C

	# of $Ar^{-/-}$ male	# of $Ar^{+/-}$ female
Zp3-Cas9 transgenic mouse embryo injected with Ar sgRNAs	15	8
C57BL/6J mouse embryo injected with Cas9 mRNA and Ar sgRNAs	9	4

S1 Fig. Sex identification using PCR with a set of primers for Sry gene. A, PCR products from C57BL/6J mouse injected with Cas9 mRNA and Ar sgRNAs. B, PCR products from Zp3-Cas9 mouse injected with Ar sgRNAs. Asterisk marks the mutant founder mice. C, knock out efficiency between two genders.
