# Supplementary material for: Generation of an Oocyte-Specific Cas9 Transgenic Mouse for Genome Editing
Source: PLoS One. 2016 Apr 27;11(4):e0154364. doi: 10.1371/journal.pone.0154364 (PMC4847922; doi:10.1371/journal.pone.0154364)

S1 Sequencing result. Sequencing results from Zp3-Cas9 transgenic mouse injected with Ar sgRNAs

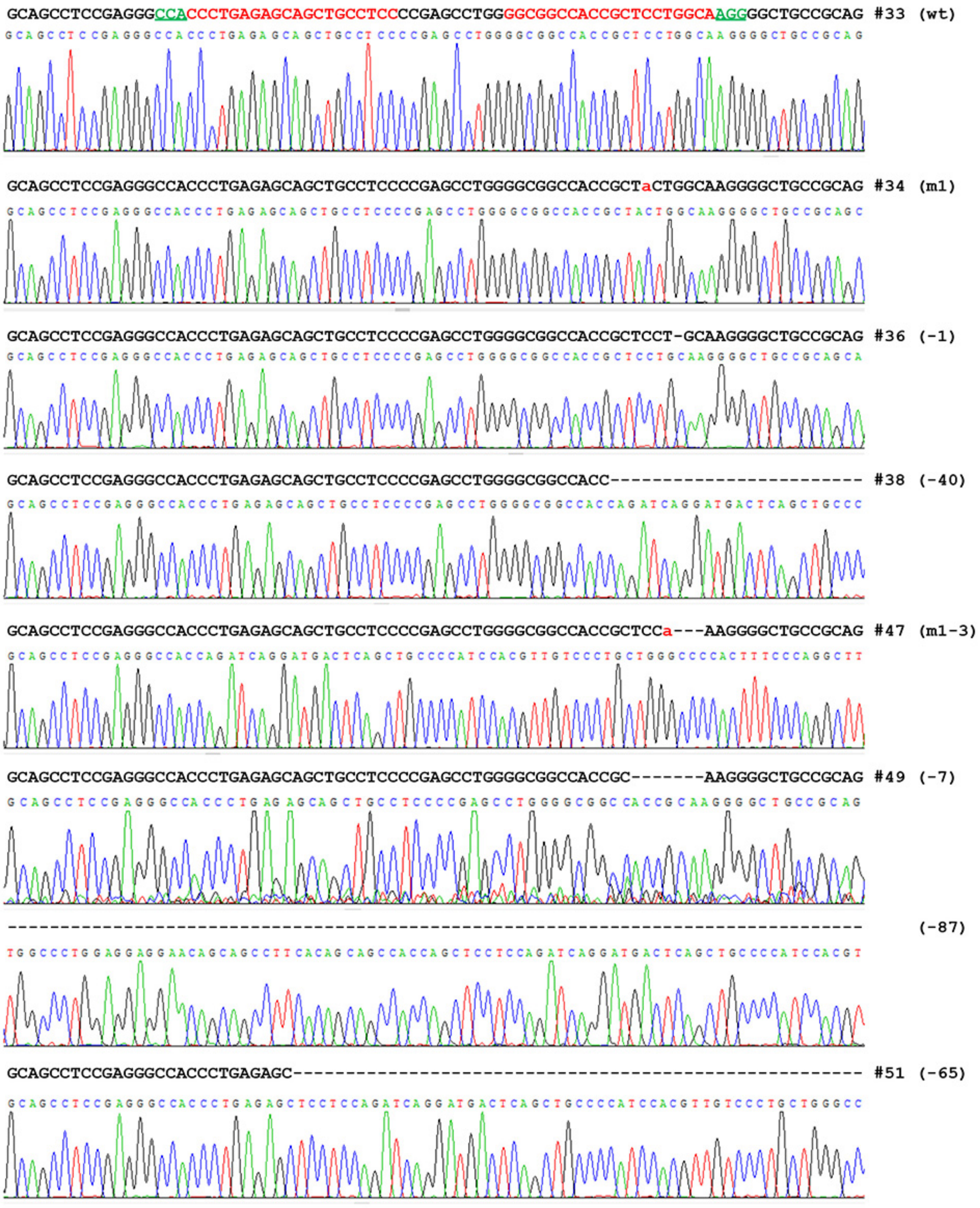

S1 Sequencing result. Sequencing results from Zp3-Cas9 transgenic mouse injected with Ar sgRNAs (continue)

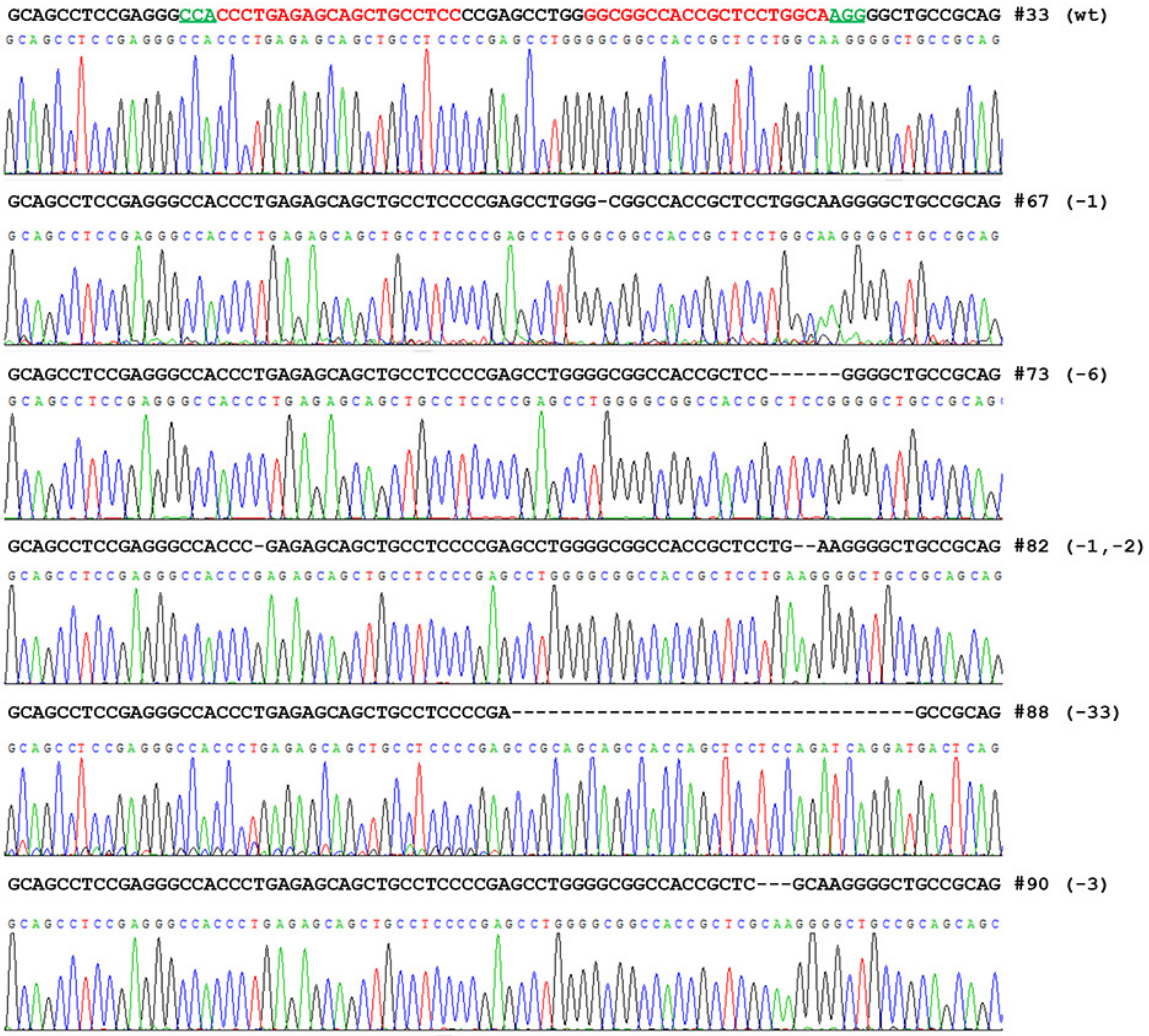

Supplement: S1 Sequencing Result — (PDF) [file pone.0154364.s002.pdf]
