# Supplementary material for: Generation of an Oocyte-Specific Cas9 Transgenic Mouse for Genome Editing
Source: PLoS One. 2016 Apr 27;11(4):e0154364. doi: 10.1371/journal.pone.0154364 (PMC4847922; doi:10.1371/journal.pone.0154364)

S2 Sequencing result. Sequencing results from C57BL/6J mouse injected with Cas9 mRNA and Ar sgRNAs

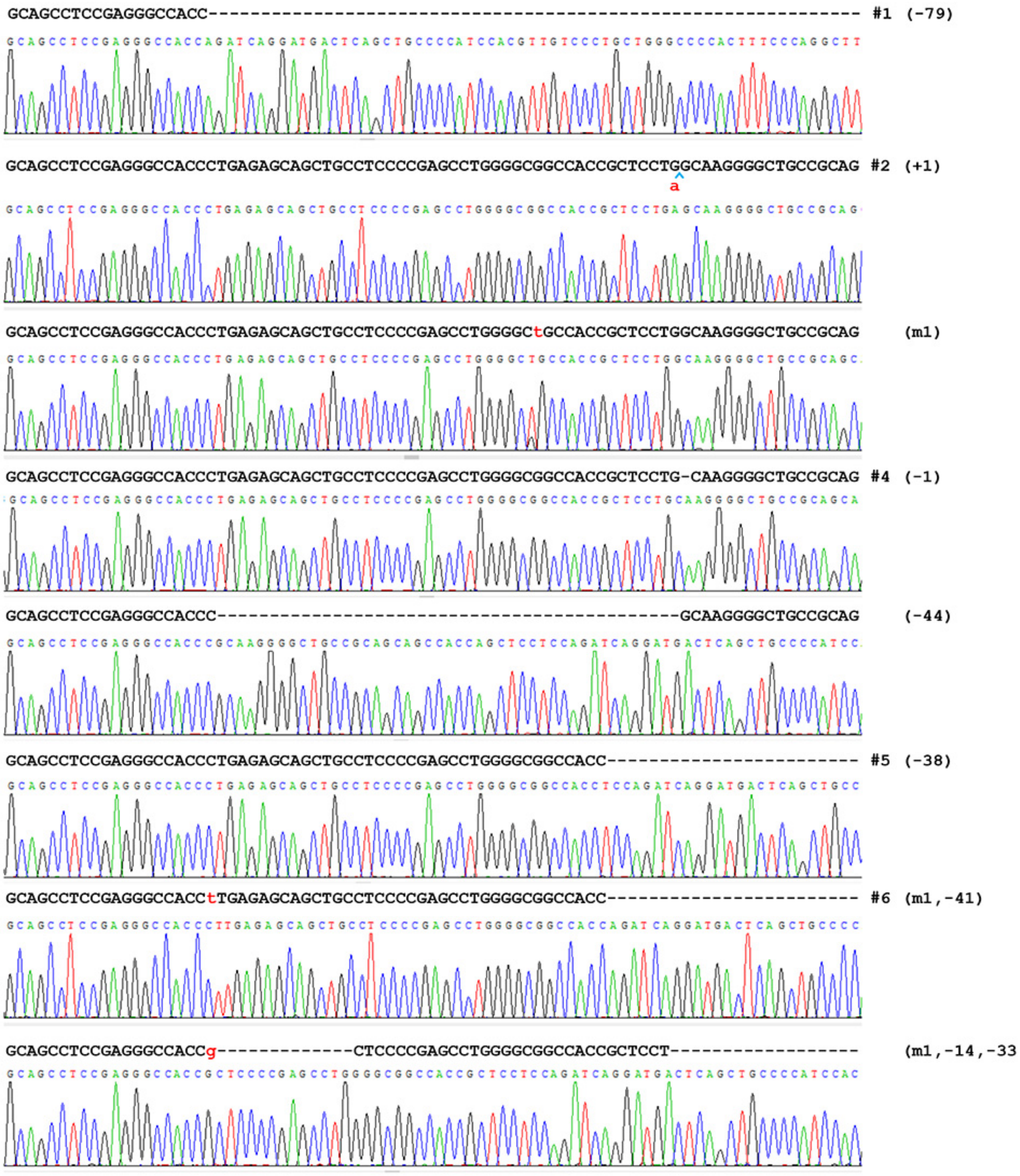

S2 Sequencing result. Sequencing results from C57BL/6J mouse injected with Cas9 mRNA and Ar sgRNAs (continue)

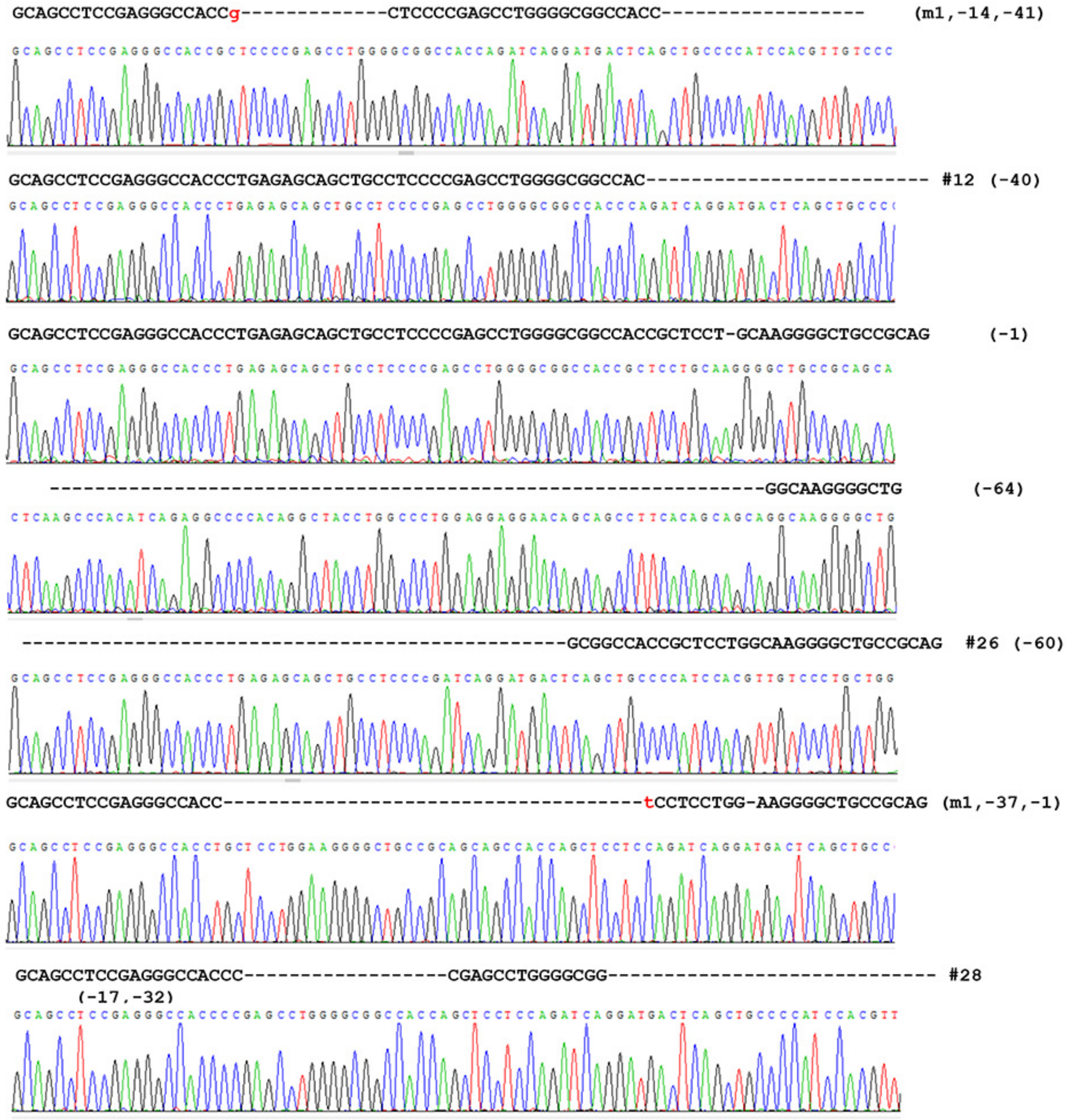

Supplement: S2 Sequencing Result — (PDF) [file pone.0154364.s003.pdf]
