# Supplementary material for: Generation of an Oocyte-Specific Cas9 Transgenic Mouse for Genome Editing
Source: PLoS One. 2016 Apr 27;11(4):e0154364. doi: 10.1371/journal.pone.0154364 (PMC4847922; doi:10.1371/journal.pone.0154364)
Supplement: S1 Table — (PDF) [file pone.0154364.s004.pdf]

S1 Table. Summary of mutant alleles with sgRNA:Cas9-mediated modifications in Zp3-Cas9 transgenic mouse embryo injection sgRNAs on AR locus

Blastocyst No.	Indels	mutants isoforms	Blastocyst No.	Indels	mutants isoforms
25	C-A	1	58	-1 bp	1
	WT			WT	
27	-1 bp	1	64	-6 bp	1
	WT			WT	
29	-4 bp	1	73	-3 bp	1
	WT			WT	
38	T-A,-3 bp	1	79	-33 bp	1
	WT			WT	
40	-7 bp	2	81	-3 bp	1
	-87 bp			WT	
	WT		89	-41 bp	1
42	-20 bp	1		WT	
	WT		99	-1 bp	1
				WT	
