# Supplementary material for: Generation of an Oocyte-Specific Cas9 Transgenic Mouse for Genome Editing
Source: PLoS One. 2016 Apr 27;11(4):e0154364. doi: 10.1371/journal.pone.0154364 (PMC4847922; doi:10.1371/journal.pone.0154364)
Supplement: S2 Table — (PDF) [file pone.0154364.s005.pdf]

S2 Table. The oligonucleotides for generating sgRNA expression

Primers	Primer sequence
AR-A-For	5’-TAGGCGGCCACCGCTCCTGGCA
AR-A-Rev	5’-AAACTGCCAGGAGCGGTGGCCG
AR-B-For	5’-TAGGAGGCAGCTGCTCTCAGGG
AR-B-Rev	5’-AAACCCCTGAGAGCAGCTGCCT
NLRP3-A-For	5’-TAGGCGGGAAGATTATGTTGGAC
NLRP3-A-Rev	5’-AAACAGTCCACATAATCTTCCCG
NLRP3-B-For	5’-TAGGCGTTCTCTGAGGCCAAAGG
NLRP3-B-Rev	5’-AAACTCCTTTGGCCTCAGAGAACG
