# Supplementary material for: Generation of an Oocyte-Specific Cas9 Transgenic Mouse for Genome Editing
Source: PLoS One. 2016 Apr 27;11(4):e0154364. doi: 10.1371/journal.pone.0154364 (PMC4847922; doi:10.1371/journal.pone.0154364)
Supplement: S3 Table — (PDF) [file pone.0154364.s006.pdf]

S3 Table. The primers for genotyping, amplifying of Cas9/sgRNA targeted fragment and sex identification

| Primers | Primer sequence | Amplicon (bp) |
|---|---|---|
| Zp3 For | 5'-GTGGTGGAATGCCTTTAATG | 662 bp |
| Cas9 Rev | 5'-GATGAGCCTCAGGTCTGCT | |
| AR out For | 5'-CTACATCATCAGTCAGGAGAACTC | 675 bp |
| AR out Rev | 5'-ATTACCTCCTGCTGCTGTTGGTG | |
| AR in For | 5'-AGGAGGCAGGATAAGGGAATTCG | 574 bp |
| AR in Rev | 5'-GCTGCTGAAGAAGTTGCATGGTG | |
| NLRP3 out For | 5'-CTACATCATCAGTCAGGAGAACTC | 675 bp |
| NLRP3 out Rev | 5'-ATTACCTCCTGCTGCTGTTGGTG | |
| NLRP3 in For | 5'-AGGAGGCAGGATAAGGGAATTCG | 675 bp |
| NLRP3 in Rev | 5'-GCTGCTGAAGAAGTTGCATGGTG | |
| Sry For | 5'-TTGTCTAGAGAGCATGGACCATGT | 273 bp |
| Sry Rev | 5'-CCACTCCTCTGTGACACTCTGCCG | |
