# Supplementary material for: Generation of an Oocyte-Specific Cas9 Transgenic Mouse for Genome Editing
Source: PLoS One. 2016 Apr 27;11(4):e0154364. doi: 10.1371/journal.pone.0154364 (PMC4847922; doi:10.1371/journal.pone.0154364)
Supplement: S4 Table — (PDF) [file pone.0154364.s007.pdf]

S4 Table. Primers for PCR amplification of the off-target sites

Off-target sites	Primer sequence		Amplicon (bp)
OTS1	Out	5’-GTCTGAGTGGTCAGATTTCC	465 bp
		5’-TACATCACAAGGAAGACCAA	
	In	5’- ATGGCTGGATATGTTTGAGA	409 bp
		5’-TACATCACAAGGAAGACCAA	
OTS2	Out	5’-TCTCTGTGTAGCCCTGACTG	595 bp
		5’-TGAGAGCTATGACTATTGGTG	
	In	5’-AACTCACTCTGTAGACCAGGCT	478 bp
		5’-AGACAGTTTCCATTACAACGCT	
OTS3	Out	5’-TTAGTGATCGCGTGCAAGGT	447 bp
		5’-CTGTGTTGGTTTCGGGCTTG	
	In	5’- AAGACTCGCCTGTGGAGTTG	339 bp
		5’-TAATACGACTCACTATAGGGG	
OTS4	Out	5’-AAGAGGAACGCGTATCTGGG	634 bp
		5’-TGAACCGTCCGCTTTAGCTT	
	In	5’-AGCAGTCTGGAGCGAGTCTT	529 bp
		5’-GCTTTAGCTTCATGCGCCG	
OTS5	Out	5’-TTGCTCAGAGACAATTTTGCAT	563 bp
		5’-GCTCTGTTCTGCAGGGTTTTG	
	In	5’- AGATGCAAAATTGTCTCTGAGC	519 bp
		5’-GGGCAAAGAGTTCGCCAAAG	
OTS6	Out	5’-AACAGTCGGGCCTGTTTTCA	525 bp
		5’-TGAGAGCTATGACTATTGGTG	
	In	5’-AGCTGGTGTCTGTTCAGTGG	438 bp
		5’-CCATTCCTGTATGGCGCTCT	
OTS7	Out	5’-AGAAGTGAACTCGCAGGACG	500 bp
		5’-CGTTGAAAGTCAGTCGGGGA	
	In	5’- ATGGCTGGATATGTTTGAGA	422 bp
		5’-TACATCACAAGGAAGACCAA	
OTS8	Out	5’-TCTCTGTGTAGCCCTGACTG	551 bp
		5’-TCCCCGACTGACTTTCAACG	
	In	5’-AACTCACTCTGTAGACCAGGCT	484 bp
		5’-GGAGTACGGCGGTTTCTGAT	

Table S4. Primers for PCR amplification of the off-target sites (Continue)

Off-target sites	Primer sequence		Amplicon (bp)
OTS9	Out	5'-CCGTCGGTGGCTCTTATTCC	567 bp
		5'-TCCAACCATTTTTGCCTGCT	
	In	5'- GTGTGAACTGCGGCTCAATC	509 bp
		5'-GCATTCAGACAAGGAAACG	
OTS10	Out	5'-CTTGACCCTGCCGACCAATA	625 bp
		5'-GCAAGCTCAACGCCGAACAA	
	In	5'-AGTGCATTGACGATGAAGAG	578 bp
		5'-ATTACATTCATCCACTGCGC	
OTS11	Out	5'-TCCTTTACAGCGAGAAATAC	457 bp
		5'-GCCGAAAAGTAGCCTAGTTT	
	In	5'- TCAAGTAGCAACAAAGAGG	409 bp
		5'-AAGTTTGGACTGAACCACT	
OTS12	Out	5'-TCTCTGTGTAGCCCTGACTG	513 bp
		5'-ACACGTTCTCACAAAAACAC	
	In	5'-TCCCCGGTGGACAGTCCTGG	465 bp
		5'-AGACAGTTTCCATTACAACGCT	
OTS13	Out	5'-GTCTGAGTGGTCAGATTTCC	433 bp
		5'-TCGCATTGTTCAGGACATTC	
	In	5'- ATGGCTGGATATGTTTGAGA	401 bp
		5'-TACATCACAAGGAAGACCAA	
OTS14	Out	5'-CTTGTAAACGGGTACTGAC	398 bp
		5'-TGAGAGCTATGACTATTGGTG	
	In	5'-GGAAACCACCAAAACTCTC	312 bp
		5'-AGACAGTTTCCATTACAACGCT	
OTS15	Out	5'-TCACTGAACCCCTCTATGC	490 bp
		5'-CTGTTTGTGGTTCCTCACAG	
	In	5'- ATGGCTGGATATGTTTGAGA	431 bp
		5'-TACATCACAAGGAAGACCAA	
OTS16	Out	5'-TCTCTGTGTAGCCCTGACTG	589 bp
		5'-GGTTCCTCACAGGAGGATGTT	
	In	5'-ACTATGAGTTGTCCTTGTGTG	540 bp
		5'-TAGTCCTGCAGCAGATGAAGTC	
